# Association of serum Clara cell protein CC16 with respiratory infections and immune response to respiratory pathogens in elite athletes

**DOI:** 10.1186/1465-9921-15-45

**Published:** 2014-04-15

**Authors:** Marcin Kurowski, Janusz Jurczyk, Marzanna Jarzębska, Sylwia Moskwa, Joanna S Makowska, Hubert Krysztofiak, Marek L Kowalski

**Affiliations:** 1 Department of Immunology, Rheumatology and Allergy, Medical University of Łódź, ul. Pomorska 251, bud. C-5, Łódź 92-213, Poland; 2 National Centre for Sports Medicine (COMS), Warsaw, Poland

**Keywords:** Respiratory viruses, Clara cell protein, Club cell protein, Exercise training, Asthma, Allergy

## Abstract

**Background:**

Respiratory epithelium integrity impairment caused by intensive exercise may lead to exercise-induced bronchoconstriction. Clara cell protein (CC16) has anti-inflammatory properties and its serum level reflects changes in epithelium integrity and airway inflammation. This study aimed to investigate serum CC16 in elite athletes and to seek associations of CC16 with asthma or allergy, respiratory tract infections (RTIs) and immune response to respiratory pathogens.

**Methods:**

The study was performed in 203 Olympic athletes. Control groups comprised 53 healthy subjects and 49 mild allergic asthmatics. Serum levels of CC16 and IgG against respiratory viruses and Mycoplasma pneumoniae were assessed. Allergy questionnaire for athletes was used to determine symptoms and exercise pattern. Current versions of ARIA and GINA guidelines were used when diagnosing allergic rhinitis and asthma, respectively.

**Results:**

Asthma was diagnosed in 13.3% athletes, of whom 55.6% had concomitant allergic rhinitis. Allergic rhinitis without asthma was diagnosed in 14.8% of athletes. Mean CC16 concentration was significantly lower in athletes versus healthy controls and mild asthmatics. Athletes reporting frequent RTIs had significantly lower serum CC16 and the risk of frequent RTIs was more than 2-fold higher in athletes with low serum CC16 (defined as equal to or less than 4.99 ng/ml). Athletes had significantly higher anti-adenovirus IgG than healthy controls while only non-atopic athletes had anti-parainfluenza virus IgG significantly lower than controls. In all athletes weak correlation of serum CC16 and anti-parainfluenza virus IgG was present (R = 0.20, p < 0.01). In atopic athletes a weak positive correlations of CC16 with IgG specific for respiratory syncytial virus (R = 0.29, p = 0.009), parainfluenza virus (R = 0.31, p = 0.01) and adenovirus (R = 0.27, p = 0.02) were seen as well.

**Conclusions:**

Regular high-load exercise is associated with decrease in serum CC16 levels. Athletes with decreased CC16 are more susceptible to respiratory infections. Atopy may be an additional factor modifying susceptibility to infections in subjects performing regular high-load exercise.

## Background

Strenuous exercise characteristic of endurance sports is believed to have a detrimental effect on the integrity of the structure of the respiratory epithelium. Such damage may lead to increased bronchial hyperresponsiveness to non-specific stimuli and contribute to the increased prevalence of exercise-induced bronchoconsriction observed in elite endurance athletes [1-4]. Endurance sports may be also associated with increased susceptibility to respiratory infections [5], which in turn, may exert a destructive effect on the airway epithelium.

Various proteins have been assessed as potentially useful tools in monitoring airway inflammation and epithelial damage [6-9]. Clara cell secretory protein (CC16), a protein secreted by non-ciliated cells of the bronchioles, has also been studied as an indicator of epithelial barrier disruption in the lower airways. Significant efforts have been undertaken to explain its role in airway inflammation and, although its function has not been fully elucidated, CC16 is known to have anti-inflammatory and anti-oxidative properties [10]. However, CC16 levels in serum depend not only on its production by Clara cells and possible leakage through the disrupted epithelial barrier, but also on renal clearance. Moreover, multiple factors may influence final CC16 serum concentration (e.g., diurnal variation, age, ethnicity, atmospheric conditions, allergen exposure, exercise). As a result of these concerns the utility of serum CC16 level measurements in monitoring of the activity of pulmonary disease, airway inflammation and chronic or acute epithelial damage is disputed (reviewed by LaKind et al. [11]).

Changes in serum CC16 levels were observed after acute, short-lasting exposure to exercise or provocative or irritating stimuli. Non-specific challenges with exercise and eucapnic voluntary hyperpnea (EVH) resulted in increased urinary CC16 levels [12,13]. In adult swimmers, exposure to trichloramine, a by-product of swimming pool disinfection, caused transient increase in serum CC16 levels while no such changes were observed in children [14].

Chronic airway inflammation may also be associated with shifts in local and systemic presence of CC16. During pollen season, CC16 in nasal lavage fluid is decreased in subjects with birch pollen-induced allergic rhinitis [15]. Decreases in serum CC16 levels in asthmatics are independent of their atopic status and correlate positively with duration of disease [16]. CC16 is believed to have not only immunomodulatory and anti-inflammatory properties but also to possibly influence the anti-infectious response: an increased persistence of viruses and prolonged viral-specific gene expression were observed in CC16-deficient mice in response to RSV infection [17].

The aim of this study was to investigate the airway epithelium inflammation and potential damage in competitive athletes, as reflected by changes in serum CC16 levels. Secondly, we sought to identify possible associations of serum CC16 level with pattern of exercise, asthma or allergy, frequent respiratory infections and the immune response to respiratory pathogens reflected by pathogen-specific IgG levels in the serum.

## Materials and methods

### Subjects

The study group consisted of 203 athletes (142 males and 61 females) preparing for the Olympic Games in Beijing in 2008 who were included into Allergy and Asthma in Polish Olympic Athletes study (A^2^POLO) which was a part of the multicentre Olympic Study performed within the framework of GA^2^LEN (Global Allergy and Asthma European Network of Excellence project). One hundred and forty-six (71.9%) of the recruited athletes were eventually included into the Polish Olympic Team for the Beijing games. The athletes represented 21 sport disciplines which, according to the predominant pattern of exercise, were divided into endurance (80 athletes) and non-endurance (123 athletes) (Table 1).

**Table 1:** Division of athletes into endurance and non-endurance group based on sport and discipline

**Sport**	**Number of athletes**
***Endurance sports (n = 80)***	
Cycling, mountain biking	11
Rowing	20
Canoe, kayak	33
Modern pentathlon	8
Triathlon	3
Swimming	5
***Non-Endurance sports (n = 123)***	
Volleyball	19
Handball	22
Weightlifting	8
Fencing	8
Gymnastics	2
Badminton	5
Shooting	6
Judo	7
Boxing	2
Wrestling	3
Track and field *(except triathlon and modern pentathlon)*	14
Archery	7
Tennis	6
Sailing	10
Windsurfing	4

The protocol of the study was approved by the Bioethics Commission of the Medical University of Lodz. All subjects gave written informed consent for participation.

Bronchial asthma and allergic rhinitis were diagnosed based on criteria published in their respective Global Initative for Asthma (GINA) and Allergic Rhinitis and its Impact on Asthma (ARIA) guidelines [18,19].

Two control groups of subjects who had never performed sport at a competitive level were recruited as a reference for serum concentrations of lung specific protein. The first group consisted of 53 healthy subjects without history of allergy or any pulmonary condition whereas the second control group comprised 49 allergic non-smoking mild asthmatics with well controlled symptoms, diagnosed according to GINA [18] and receiving anti-inflammatory treatment on a permanent basis. More detailed characteristics of the study and control groups are shown in Tables 2 and 3.

**Table 2:** Demographics and lung function parameters in healthy and asthmatic control subjects and in athletes

	**Healthy controls**	**Asthma controls**	**All athletes**	**Endurance athletes**	**Non-endurance athletes**
**No. of subjects**	53	49	203	80	123
**Males/Females**	23/30	15/34	142/61	60/20	82/41
**Age[years] (median; range)**	28 (20-45)	29 (18-45)	26 (18-41)	25	26
**Baseline FEV1 [% predicted] (median;range)**	110 (77-126)	100 (75-123)	103 (66-129)	106 (82-126)	100 (66-129)
**ΔFEV1post-salbutamol [%] (median; range)**	ND	ND	4 (0-26)	4 (0-20)	4 (0-26)
**MEF 25-75 [% predicted] (median; range)**	104	80^*^	97 (38-170)	96 (42-170)	98 (38-163)

^*^p < 0.05 vs. HC, all athletes, endurance athletes and non-endurance athletes;; ND, not done; HC, healthy controls

**Table 3:** Demographics and lung function parameters in healthy and allergic athletes

	**Athletes with rhinitis**	**Athletes with asthma**	**Athletes without asthma or allergy**
**No. of subjects**	30	27	146
**Males/Females**	19/11	17/10	97/41
**Age[years] (median; range)**	26	25	26
**Baseline FEV1 [% predicted] (median;range)**	105 (80 – 129)	99 (66 – 117)	103 (81 – 125)
**ΔFEV1post-salbutamol [%] (median; range)**	4 (0 – 10)	10.5 (2 – 26)^a^	4 (0 – 20)
**FVC [% predicted] (median; range)**	101 (92 – 120)	108 (77 – 125)	106.5 (85 – 134)
**FEV1/FVC (median; range)**	0.81 (0.72 – 0.92)	0.77 (0.63 – 0.86)	0.81 (0.61 – 0.97)
**MEF 25-75 [% predicted] (median; range)**	98 (54 – 156)	82 (38 – 122)^b^	99 (42 – 170)

^a^p < 0.001 vs. athletes with rhinitis and athletes without asthma or allergy;

^b^p < 0.05 vs. athletes with rhinitis and athletes without asthma or allergy.

The following subgroups of athletes were distinguished for the purpose of further analysis: athletes diagnosed with allergic rhinitis (n = 30); athletes diagnosed with allergic asthma with or without concomitant rhinitis (n = 27); and athletes without asthma or allergy (n = 146), basing on the diagnosis established during clinical evaluation of athletes. The data from an Allergy Questionnaire for Athletes (AQUA) were analysed separately.

### AQUA questionnaire

Data regarding type of sport, allergy symptoms and history of respiratory infections, as well as demographical data, were acquired from the Allergy Questionnaire in Athletes (AQUA©) which was filled by each athlete entering the study. The AQUA questionnaire, developed and validated by Bonini et al [20] in Italian soccer players, is intended as an allergy screening tool in competitive athletes, with AQUA© score values ≥5 described as having the best positive predictive value for allergy. The questionnaire is protected by copyright and its use in this study was kindly permitted by the authors.

### Lung function tests

Spirometry was performed according to criteria defined by a joint ERS/ATS task force [21] using a Lungtest 1000 spirometer (MES, Kraków, Poland). Subjects receiving antiasthmatic treatment were asked to suspend taking long-acting beta2-agonists for 24 hours, short-acting beta2-agonists for 12 hours, inhaled steroids for 7 days and leukotriene modifiers for 5 days before lung function testing. At least three measurements in the upright position were performed and the best result was recorded. Reversibility test with bronchodilator was performed only in athletes. For this purpose, 400 μg of salbutamol was administered by metered dose inhaler, followed by spirometry measurement 15 minutes later. Changes in forced expiratory volume in 1 s (ΔFEV1) from before to after drug inhalation were calculated according to the formula: 100 × (postsalbutamol FEV1 - initial FEV1)/initial FEV1.

### Laboratory measurements

Serum samples were obtained from 86% (n = 119) non-endurance and 94% (n = 75) endurance athletes. The remaining athletes were not available for blood sampling due to training schedules. Blood was drawn into Monovette tubes for serum (Sarstedt, Landskrona, Sweden). Each sample was left to clot at room temperature for ca. 30 minutes. Subsequently, samples were centrifuged at 700 *g* for 10 minutes and serum was transferred to cryotubes and frozen at –80°C for further analyses.

CC16 concentration was determined using Human Clara Cell Protein ELISA kits (BioVendor Laboratory Medicine, Inc., Modrice, Czech Republic) in accordance with instructions provided by the manufacturer. Samples were run in duplicate at a 25-fold dilution, following the manufacturer’s recommendations. The lower CC16 detection limit is 20 pg/ml.

The serum concentration of IgG antibodies against parainfluenza virus 1,2 and 3 (PIV) was determined by commercially available ELISA kit (DRG International, Inc., Mountainside, NJ, USA). The threshold value for positive anti-PIV IgG testing was 10 U/ml.

The serum concentrations of IgG against respiratory syncytial virus (RSV), adenovirus and *Mycoplasma pneumoniae* were determined by commercially available semi-quantitative ELISA kits (Orgenium Laboratories, Vantaa, Finland) The threshold value for positive testing was 25 Enzyme Immunoassay Units (EIU).

Concentrations of serum IgG’s against repiratory pathogens were determined in athletes and healthy control groups only.

### Statistics

Statistical analyses were performed using Statistica (Statsoft Polska, Krakow, Poland) and GraphPad Prism (Graphpad Software, Inc., La Jolla, CA, USA) software. The Kolmogorov-Smirnov test with Lilliefors correction was used to check for normality of values’ distribution. To identify differences between groups, the Mann-Whitney *U* test or Kruskal-Wallis test with Dunn’s *post hoc* test were used where applicable. Correlations of age, total IgE, lung function parameters, reversibility challenge results and levels of IgG against respiratory pathogens with serum CC16 level were assessed with Spearman’s rank correlation test.

The chi-square test with Yates’ correction was used for between-the-group comparisons of percentage of positive tests for IgG against the presence of PIV, RSV, adenovirus or *M. pneumoniae*.

Relationships between serum CC16 level and AQUA score value with the presence of asthma/allergy symptoms and frequent respiratory infections were analysed using logistic regression.

A *P* value less than 0.05 was considered statistically significant in all analyses.

## Results

### Clinical features

Upon clinical evaluation, asthma was diagnosed in 27 athletes (13.3%) of whom 15 (55.6%) had concomitant allergic rhinitis, whereas allergic rhinitis (without concomitant asthma) - in 30 subjects (14.8%).

An affirmative answer to the question “Do you frequently suffer from upper respiratory infections (pharyngitis, bronchitis, colds) or fever?” was given by 39 athletes (19.2%). No differences in the prevalence of self-reported frequent infections were seen between atopic and non atopic athletes (20.1% vs. 18.4%; p > 0.05, *χ*^2^ test).

Lung function parameters did not differ significantly between endurance and non-endurance athletes. No differences were observed between athletes (endurance and non-endurance) and any of the control groups – Table 2. The median FEV1, FVC and FEV1/FVC values in asthmatic athletes were not significantly different from those observed in athletes with rhinitis and in non-asthmatic non-allergic athletes. Median MEF25-75 values were significantly lower in athletes with asthma as compared to other athletes. Also, MEF25-75 values were significantly reduced in asthmatic controls compared to the healthy controls and the athletes – Table 2. The percentage increase in FEV1 after salbutamol was significantly higher in athletes with asthma than in non-asthmatics (10.5% vs 4%; p < 0.001) – Table 3.

### Serum CC16 and exercise pattern

**Figure 1:**
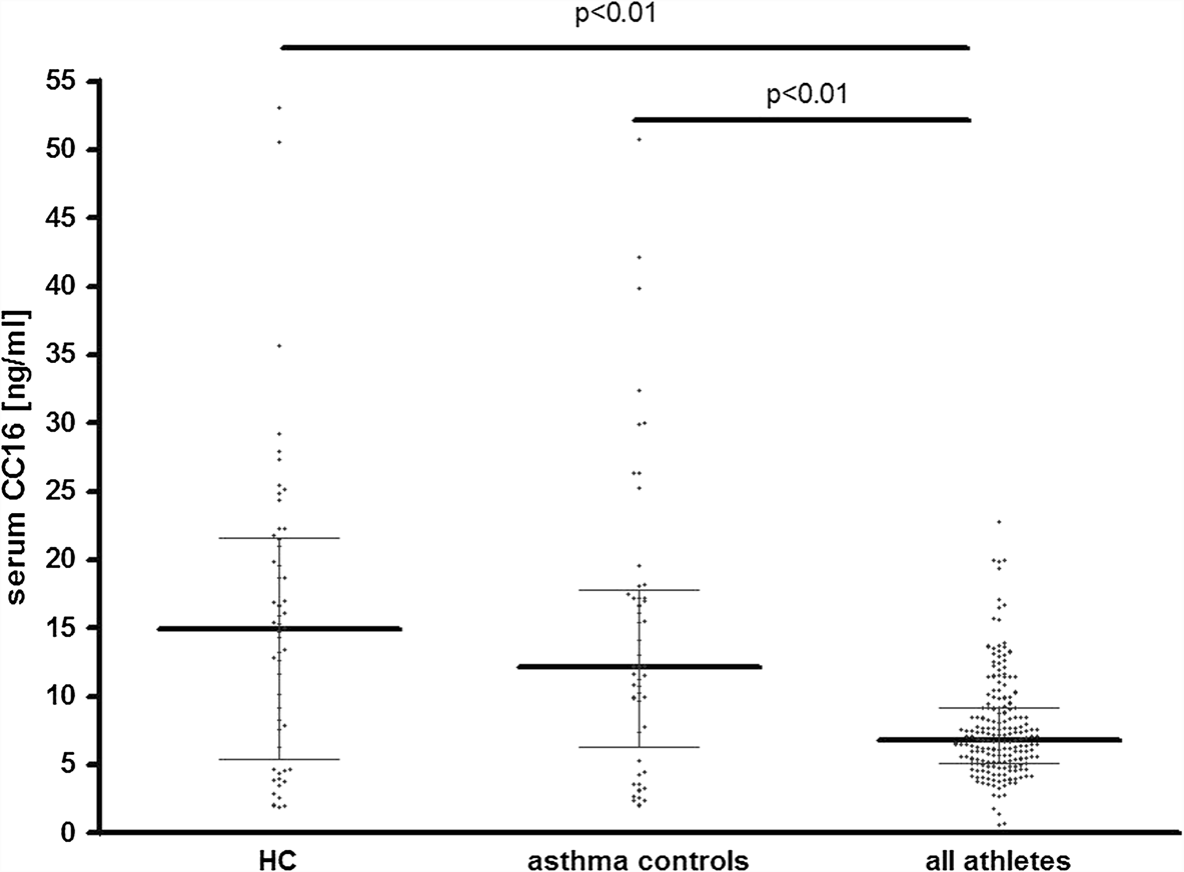
**Serum Clara cell protein (CC16) concentration in healthy controls (HC), mild asthmatic controls and in athletes.** One dot represents one subject. Medians with interquartile ranges are marked with horizontal lines.

Serum CC16 concentration was measured in 75 endurance and 123 non-endurance athletes and in two control groups of non-athletes. The mean serum CC16 concentration was significantly lower in the “all-athletes” group than in healthy controls and asthmatic controls – Figure 1. No differences in the serum CC16 concentrations were observed between endurance and non-endurance athletes (7.13 [5.09-9.43] vs. 6.67 [5.20-9.14] ng/ml; medians with interquartile ranges, p > 0.05]). Considering possible noxious effect of trichloramines on airway epithelium and Clara cell function, additional analysis was performed to assess possible influence of competitive swimming on serum CC16 levels. Exclusion of swimmers and triathletes (n = 8) did not significantly influence the serum CC16 level (6.79 [5.17-9.18] ng/ml without swimmers and triathletes vs. 6.82 [5.14-9.17] ng/ml in all athletes; medians with interquartile ranges, p > 0.05, Mann-Whitney *U* test).

### Serum CC16, allergic disease and respiratory infections

**Figure 2:**
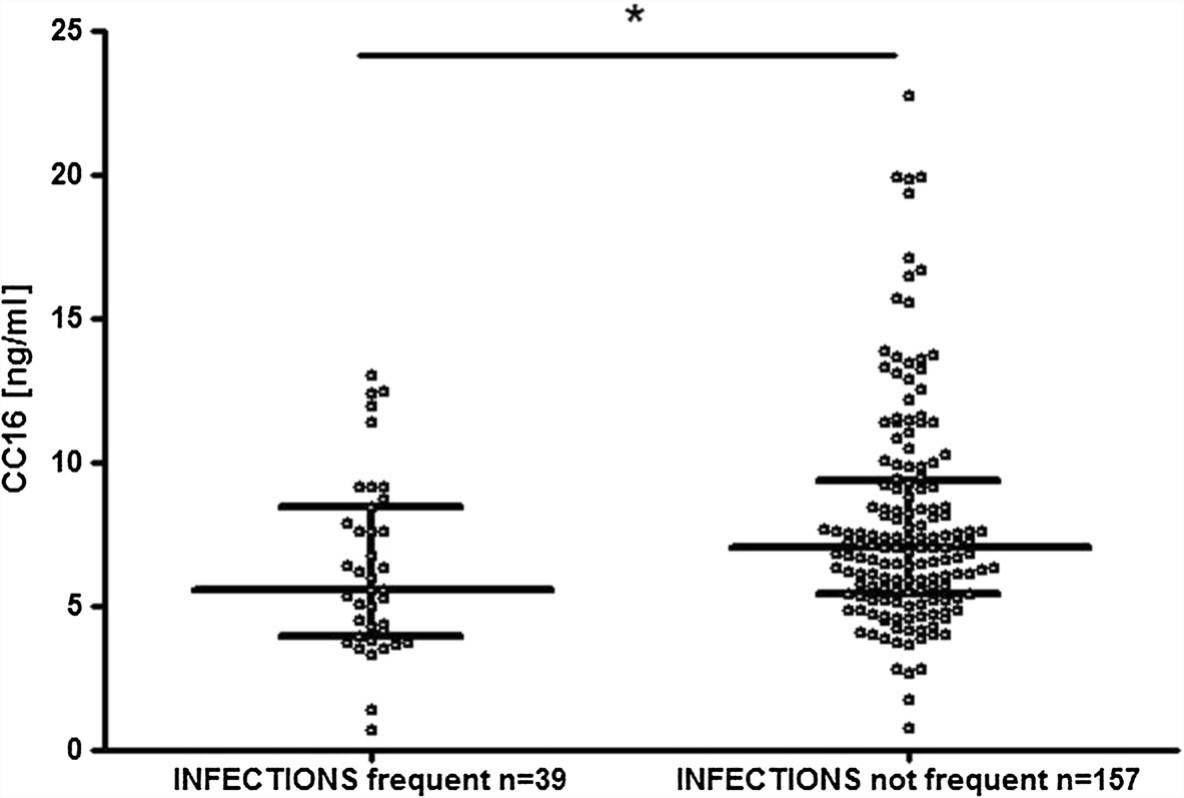
**Serum CC16 levels in athletes reporting and not reporting frequent respiratory infections when completing the AQUA© questionnaire.** Medians with interquartile ranges are marked with horizontal lines. One dot represents one subject. * p = 0.01, Mann Whitney *U* test.

Levels of serum CC16 did not differ significantly between atopic and non-atopic athletes (6.88 [5.37-9.15] vs. 6.68 [5.03-9.06] ng/ml; medians with interquartile ranges, p > 0.05]). No association was found between CC16 levels and self-reported asthma symptoms or between serum CC16 and physician-diagnosed asthma or allergic rhinitis. However, in athletes reporting frequent upper respiratory infections, the median serum CC16 levels were significantly lower than in the remaining athletes (median values 5.57 vs 7.03 ng/ml; respectively, p = 0.01, Mann-Whitney *U* test) – Figure 2.

No significant correlation of serum CC16 with asthma, baseline FEV1 or percentage of airway reversibility after beta-agonist treatment was found in the all-athletes group or in any of the athlete subgroups: ie, endurance, non-endurance, allergic/asthmatic or healthy athletes. Similarly, no correlations of serum CC16 with age or lung function parameters were found in the control groups.

Logistic regression analysis using ‘serum CC16 ≤ 4.99 ng/ml’ (considered as the low value) as an independent variable showed that the risk of frequent URTIs, as reported in the AQUA questionnaire, was significantly higher in athletes with low serum CC16 (OR = 2.56; 95%CI:1.19-5.50; p < 0.02). Also, the risk of declared nasal symptoms, not identical with physician-diagnosed rhinitis, was higher in athletes with serum CC16 ≤ 4.99 ng/ml (OR = 2.21; 95% CI:1.10-4.47; p < 0.03).

No association was found between a serum CC16 level lower than 4.99 ng/ml and diagnosis of allergic rhinitis, diagnosis of asthma, declared cough and/or dyspnoea without physician-confirmed asthma, presence of atopy or positive serological test for any respiratory pathogen. Logistic regression analysis using ‘serum CC16 ≤ 4.99 ng/ml’ as a dependent variable did not show decreased serum CC16 levels to be determined either by predominant endurance exercise nor by length of the single training session.

### Serum IgG immune response to respiratory pathogens

The prevalence of positive serological testing for anti-PIV, anti-RSV or and anti- *M.pneumoniae* (but not for antiadenovirus) antibody, was significantly lower among athletes than among healthy subjects. The mean serum concentration of Anti-PIV IgG levels was significantly lower, whereas concentration of anti-AdV IgG was significantly higher in professional athletes - Table 4.

**Table 4:** Percentage of positive test results and levels of IgG against respiratory pathogens in healthy controls (HC) and olympic-level athletes

**Pathogen**	**HC**	**Athletes**	**P value [*χ***^**2 **^**test]**	**HC**	**Athletes**	**P value [Mann-Whitney *U *** **test]**
	***Percentage of positive serological tests***			***median IgG level***		
PIV 1,2,3	95.7%	74.7%	**<0.0003**	99.5 U/ml	32.9 U/ml	**<0.0001**
RSV	84.4%	68.2%	**0.01**	39.7 EIU	35.3 EIU	0.25
AdV	72.7%	71.3%	0.93	33.4 EIU	44.2 EIU	**0.0005**
*M. pneumoniae*	100%	90.2%	**0.01**	66.3 EIU	65.2 EIU	0.38

PIV, parainfluenza virus; RSV, respiratory syncytial virus; AdV, adenovirus. Significant *P* values given in bold print.

The percentage of positive serological tests and median serum levels of IgG against respiratory pathogens did not differ between athletes declaring frequent URTIs in AQUA questionnaire and the remaining group - Table 5.

**Table 5:** Percentage of positive test results and levels of IgG against respiratory pathogens in athletes with and without frequent upper respiratory tract infections (URTIs)

**Pathogen**	**Athletes with frequent URTIs**	**Athletes without frequent URTIs**	**P value [*χ***^**2 **^**test]**	**Athletes with frequent URTIs**	**Athletes without frequent URTIs**	**P value [Mann-Whitney *U *** **test]**
	***Percentage of positive serological tests***			***Median IgG level***		
PIV 1,2,3	47.2%	52.6%	0.70	25.4 U/ml	33.0 U/ml	0.17
RSV	71.8%	67.5%	0.75	32.8 EIU	35.3 EIU	0.66
AdV	66.7%	72.7%	0.58	43.9 EIU	44.3 EIU	0.66
*M. pneumoniae*	92.3%	90.9%	0.96	64.3 EIU	65.7 EIU	0.64

PIV, parainfluenza virus; RSV, respiratory syncytial virus; AdV, adenovirus.

### Correlations of serum CC16 and IgG immune response to respiratory pathogens

In all athletes, a weak yet significant positive correlation of serum CC16 and IgG concentration was observed only for PIV (R = 0.20, p < 0.01) but not for the remaining pathogens. In atopic athletes, positive correlations were observed between the levels of serum CC16 and IgG against RSV (R = 0.29, p = 0.009), PIV (R = 0.31, p = 0.01) and Adenovirus (R = 0.27, p = 0.02). No significant correlations were observed in healthy controls.

**Figure 3:**
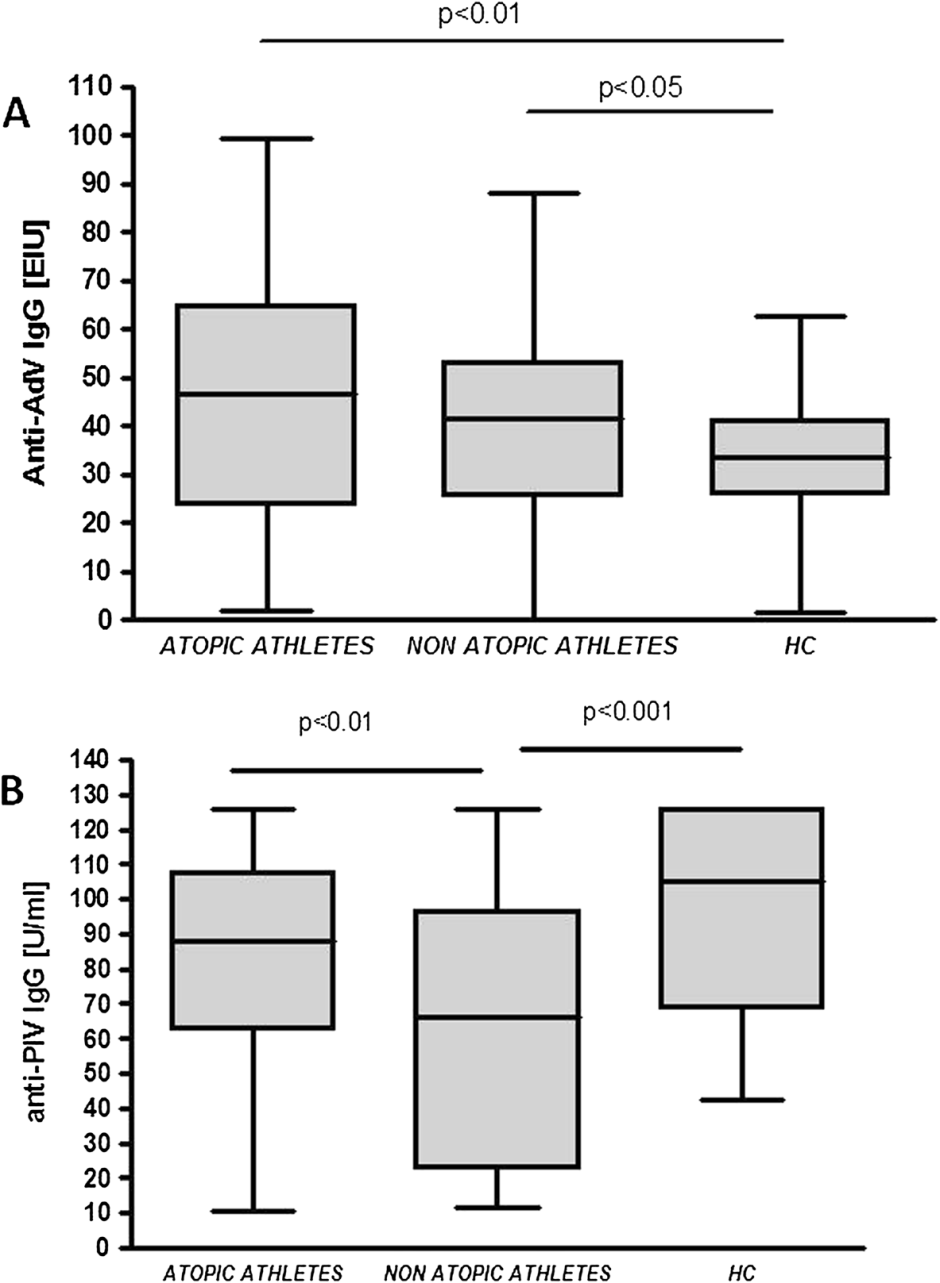
**Box and whisker plots presenting serum anti-AdV1,2,3 (panel A) and serum anti-PIV (panel B) IgG levels in athletes and healthy controls (HC).** Ends of whiskers present minimum and maximum values in each group; PIV, parainfluenza virus; AdV, adenovirus.

Higher anti-adenovirus IgG titres were found in both atopic and non-atopic athletes as compared to HC (46.4 [23.5-64.8] and 41.6 [25.4-53.3] vs 33.4 [25.8-41.2] EIU, respectively; median [interquartile range], p < 0.001) - Figure 3A. Non-atopic athletes had lower anti-PIV IgG levels than atopic athletes (65.9 [22.6-96.5] vs 88.1 [62.8-107.6] U/ml; median [interquartile range], p < 0.01), and lower than HC (65.9 [22.6-96.5] vs 104.8 [68.5-126.0] U/ml; median [interquartile range], p < 0.001) – Figure 3B. No significant differences between atopic and non-atopic athletes and HC were seen with regard to anti-RSV and anti-*Mycoplasma pneumoniae* IgG levels (data not shown).

When the results of the positive serological testing were analysed, a significantly lower percentage of subjects with positive anti-RSV serology was observed in non atopic athletes (60.8%) than in healthy controls (84.4%, p = 0.001) and atopic athletes (76.3%, p = 0.03). The presence of a positive RSV serology test was significantly associated with atopy (OR = 2.89; 95%CI, 1.34-6.23; p = 0.007). Such associations were not observed regarding serological testing for IgG against PIV, adenovirus and *M. pneumoniae* – data not shown.

## Discussion

This study documents that serum CC16 concentrations are significantly lower in top athletes than in control subjects from the general population. Decreased serum CC16 concentrations were reported in infants and children regularly visiting indoor pools [22,23] but other studies brought conflicting results regarding the effect of single and repeated training session on serum CC16 [24,25]. Discrepancies are believed to be due to intensity of exertion and breathing pattern employed during various types of athletic activity (reviewed by LaKind et al. [11]). Our study, including large and heterogeneous group has shown decreased serum CC16 levels in professional athletes, irrespective of the type or pattern of high-level activity. However, due to the limited number of athletes representing each discipline, it was impossible to assess the influence of each type of sport on serum CC16 concentration. We have not ascertained any influence of predominant exercise pattern (endurance versus non-endurance) on athletes’ serum CC16 levels. Although withdrawal of swimmers and triathletes from the serum CC16 analysis did not influence final result, these data - in our opinion – are too scarce to conclude that unfavourable training environment (e.g., trichloramine exposure) does not affect serum CC16 concentration.

CC16 is a protein of multifaceted characteristics. It may be considered both as a marker of the disruption of airway epithelial barrier integrity and as a co-participant in anti-inflammatory response. Degree of CC16 involvement and significance in the aforementioned processes is still a matter of research and dispute.

Our results suggest, that it is an intensive and regularly performed exercise *per se* that stimulates airway inflammation, irrespective of training intensity. Reduced presence of CC16, an anti-inflammatory factor, may additionally contribute to airway inflammation and hyperresponsiveness.

In addition, the control non-athletes asthmatic patients were found to have CC16 levels similar to those in healthy controls, which contrasts with the results of other report, documenting a significant decrease of serum CC16 in asthma [16]. In our opinion, the possible explanation for this discrepancy is the fact that our asthmatic controls group comprised exclusively subjects with mild disease well controlled on anti-inflammatory treatment. Therefore, it is possible that that changes in serum CC16 level reflect the grade of airway inflammation in asthma, similarly as it was observed in regard to sputum CC16 in subjects with COPD [26]. Our data suggest that the issue of CC16 in chronic asthma be extensively studied in a well-defined population.

Although recent data published by Jacobs et al [27] show that lower serum CC16 levels may be associated with allergic sensitisation, no differences in CC16 levels between atopics and non-atopics were observed in our population of athletes. Therefore, the associations of allergic sensitisation with markers of epithelial barrier disruption require further study.

Another novel observation is that athletes declaring frequent upper respiratory tract infections had significantly lower serum CC16 concentrations than those without frequent infections - Figure 2. Accordingly, a higher risk of frequent respiratory infections was associated with low serum CC16 level. Low serum CC16 was found to be also associated with patients’ reported symptoms of rhinitis, but not with physician-diagnosed rhinitis. This apparent discrepancy may be explained by the fact that symptoms declared as “rhinitis” were, in fact, symptoms of upper respiratory infections. This is congruous with observation that both frequent of URTIs and reported rhinitis symptoms are considerably more likely to be encountered in athletes with serum CC16 below 5 ng/ml.

The common belief that moderate exercise is beneficial to one’s health whereas excessive physical training may considerably increase the susceptibility to infection was confirmed by the concept of the U-shaped relationship between physical activity and resistance to disease [5]. Our study comprised top-level athletes and, although only 19.2% of them claimed to be suffering from frequent respiratory infections, those who reported infections had significantly lower serum CC16 concentrations. This interaction is further strengthened by significant associations of decreased serum CC16 levels with frequent declared respiratory infections and nasal symptoms and indicates, that the immune response against some respiratory pathogens may be modified by competitive sporting activity. Positive correlation of anti-PIV IgG and serum CC16 may be considered an indirect proof for immunomodulatory activity of the latter, but the lack of such correlation for the remaining pathogens is somewhat puzzling and suggests the need for further study of the influence of CC16 on the susceptibility to infection with particular pathogens.

Less frequent positive serological test results against PIV, RSV and *M. pneumoniae* in professional athletes compared to controls may be interpreted as indirect evidence for impaired immunocompetence resulting from continuous high exercise load. In a mouse model, CC16 was shown to influence the lymphocyte response of the airways after infection with RSV. Mice lacking CC16 demonstrated significantly higher concentrations of Th2 cytokines IL-5 and IL-13 in BALF for several days following the infection [17]. The stimulatory activity of RSV infection on Th2 cytokine synthesis and release may explain the association of atopy with more frequent occurrence of positive testing for anti-RSV IgG in atopic athletes.

The frequency of positive immune response to RSV was comparable between atopic athletes and healthy control subjects, but it was significantly lower in non-atopic athletes. In addition, correlations between serum CC16 level and intensity of immune response to respiratory viruses (RSV, PIV and adenoviruses) were only significant in atopic athletes. Given that serum CC16 levels in athletes were not seen to be modified by atopy status, it may suggest, that atopy *per se* influences the immune response to respiratory viruses.

Our study has several limitations. The study group was quite heterogeneous in terms of predominant pattern of exercise and training environment not allowing for identification of possible effects of any particular sport on CC16 or immune response against respiratory pathogens. Accordingly, no standardised and comparable measures of exercise load/intensity could be included into analysis. Furthermore, the AQUA question concerning respiratory infections does not determine their actual frequency, allowing only for the subjective assessment of whether the URTIs are frequent or not.

Another limitation of our study results from the inter-subject variability of CC16 levels. Overlaps in the serum CC16 are observed between the groups and it could be argued that outliers influence the overall result. Significant differences were shown through non-parametric tests which makes possibility of such influence less likely. However, this issue should be considered possible confounder and addressed in next studies on CC16 in larger populations.

For the purpose of logistic regression analysis, 5.0 ng/ml was set arbitrarily as the CC16 threshold value, based on the reference value given by the manufacturers of the ELISA kits. However, the CC16 levels in healthy subjects are variable and the accepted reference values for CC16 are actually lacking, which substantially hinders such analysis and signals the need for further assessment of CC16 levels in various clinical models [7,24,28].

Bronchial hyperreactivity was assessed only in few patients (due to their training schedules and low accessibility) and could not be analysed in this study. However, of the role of CC16 in the context of bronchial hyperresponsiveness may be important for understanding the pathogenesis of EIB, especially in view of the data showing association of *CC16* gene polymporphism with airway hyperresponsiveness and asthma phenotype [29].

## Conclusions

Our study demonstrated that regular high-load exercise training is associated with a decrease in serum CC16 protein levels and lower CC16 levels in competitive athletes are associated with increased prevalence of reported respiratory infections. Further studies are necessary to elucidate the mechanism of association between increased susceptibility to respiratory infections in top athletes and epithelial cell injury markers such as CC16.

## Competing interests

The authors declare they have no competing interests regarding this article.

## Authors’ contributions

MK and MLK participated in conception and design of the study, in analysis and interpretation of data, and in manuscript preparation; MK, JJ and HK participated in subjects’ recruitment and clinical assessment; MJ and SM performed laboratory measurements; JSM recruited and assessed subjects from control groups. All authors read and approved the final manuscript.
